# State of the art in the determination of trace elements in seawater: a worldwide proficiency test

**DOI:** 10.1007/s00216-016-9390-6

**Published:** 2016-02-17

**Authors:** Pieter Dehouck, Fernando Cordeiro, James Snell, Beatriz de la Calle

**Affiliations:** European Commission, Directorate General Joint Research Centre, Institute for Reference Materials and Measurements, Retieseweg 111, 2440 Geel, Belgium

**Keywords:** Proficiency test, Trace elements, Seawater, Inductively coupled plasma mass spectrometry, Inductively coupled plasma optical emission spectrometry, Atomic absorption spectroscopy

## Abstract

This manuscript presents the results of the International Measurement Evaluation Programme 40 (IMEP-40) study, a proficiency test (PT) which was organised to assess the worldwide performance of laboratories for the determination of trace elements in seawater. This PT supports the implementation of the European Union Water Framework Directive 2000/60/EC, which aims at achieving a long-term high level protection of the aquatic environment, covering lakes, ground water and coastal waters. Forty-six participants reported results. The test item was seawater containing the trace elements As, Cd, Co, Cr, Cu, Fe, Mn, Mo, Ni, Pb, Se and Zn. The trace elements in the test item were present in very low concentrations to mimic natural levels. The results of the participants were rated with *z* and zeta (*ζ*) scores in accordance with ISO 13528 and ISO 17043. The standard deviation for proficiency assessment, $\widehat{\upsigma}$, was set at 25 % of the respective assigned values for the 12 measured elements based on previous experience with similar PTs. The low levels of the trace elements combined with the high salt concentration of the seawater made the measurements challenging. Many laboratories were unable to detect or quantify the elements and reported “lower than X” values. The percentage of satisfactory performances (expressed as *z* scores) ranged from 41 % (Cr, Fe) to 86 % (Mo). The PT study showed that the use of proper standard methods, like ISO 17294-2, and sensitive techniques, like inductively coupled plasma mass spectrometry (ICP-MS), contributed to performing well in this PT round.

## Introduction

The monitoring of trace elements in seawater is relevant for the implementation of the Directive 2000/60/EC (Water Framework Directive (WFD)), which aims at achieving a long-term high level protection from chemical pollution of the aquatic environment, covering lakes, ground water and coastal waters [1]. The WFD establishes a list of priority substances. The daughter Directive 2013/39/EU [2] lays down the environmental quality standards (EQS) for priority substances and other pollutants with the aim of achieving good surface water chemical status. Regarding the trace elements investigated in this proficiency test study, maximum allowable concentrations in seawater are set for Cd (0.45 μg L^−1^), Pb (14 μg L^−1^) and Ni (34 μg L^−1^) [2]. The levels of a number of trace elements present in this study (As, Cd, Cr, Cu, Ni, Pb, Zn) are also limited by Directive 2006/113/EC on the quality required of shellfish waters [3]. This directive applies to coastal and brackish waters that need protection or improvement in order to support shellfish (bivalve and gastropod molluscs) life and growth and thus contribute to the high quality of shellfish products edible by man. Besides ensuring compliance with legislation, the monitoring of trace elements in seawater is carried out for research purposes to study the global status of trace elements in the oceans. The international GEOTRACES programme is a study of the global marine biogeochemical cycles of trace elements and their isotopes [4]. Recent research has revealed the important role of trace elements in controlling marine biogeochemical processes [5]. Trace metals such as Fe and Co are involved in the regulation of primary productivity in phytoplankton species and therefore play a role in controlling the global climate by modulating the biological uptake of CO_2_ in the ocean [6, 7].

Different techniques have been applied for the measurement of trace elements in seawater like atomic absorption spectroscopy (AAS) comprising electrothermal atomic absorption spectroscopy (ET-AAS) [8, 9], inductively coupled plasma atomic emission spectrometry (ICP-AES), also referred to as inductively coupled plasma optical emission spectrometry (ICP-OES) [10] and inductively coupled plasma mass spectrometry (ICP-MS) [11–17]. The development of highly sensitive detection methods and the use of clean sampling and handling techniques are essential in order to measure the low levels of trace elements naturally present in seawater [4]. ICP-MS has become one of the most powerful analytical techniques for the multi-element determination of trace elements [11]. However, seawater is a complex matrix with a high salt concentration which may interfere with the ICP-MS measurements of low level trace elements. The high salinity of seawater samples can cause salt precipitation and build-up in the ICP-MS instrument. Finally, polyatomic interferences formed during the ICP-MS analysis may limit the determination of trace elements in seawater. Table 1 is taken from reference [18] and shows the most abundant polyatomic interferences for the trace elements analysed in this study.

**Table 1:** Isotopes of interest and their most frequent polyatomic interferences for the analysed trace elements in seawater (taken from ref. [18])

Isotope	Interfering species
^75^As	^40^Ar^35^Cl, ^40^Ca^35^Cl
^111^Cd	^79^Br^32^S
^112^Cd	^96^Mo^16^O
^114^Cd	^98^Mo^16^O
^59^Co	^36^Ar^23^Na, ^24^Mg^35^Cl, ^42^Ca^16^OH, ^23^Na^35^ClH
^52^Cr	^36^Ar^16^O, ^40^Ar^12^C, ^35^Cl^16^OH, ^37^Cl^14^NH
^63^Cu	^40^Ar^23^Na, ^40^Ca^23^Na
^65^Cu	^40^Ar^25^Mg, ^40^Ar^24^MgH
^54^Fe	^40^Ar^14^N, ^38^Ar^16^O, ^37^Cl^16^OH, ^40^Ca^14^N
^56^Fe	^40^Ar^16^O, ^40^Ca^16^O
^55^Mn	^40^Ar^14^NH, ^40^Ar^15^N, ^39^K^16^O, ^23^Na^32^S, ^37^Cl^18^O
^98^Mo	^40^Ar^23^Na^35^Cl
^58^Ni	^40^Ar^18^O, ^23^Na^35^Cl, ^42^Ca^16^O
^60^Ni	^23^Na^37^Cl, ^25^Mg^35^Cl
^64^Zn	^40^Ar^24^Mg, ^40^Ar^23^NaH, ^32^S^16^O^16^O
^66^Zn	^40^Ar^26^Mg
^68^Zn	^40^Ar^14^N_2_

To minimise these interferences, many methods use a pre-concentration step prior to detection. Different pre-concentration techniques for trace elements in seawater have been described including solid phase extraction (SPE) using metal affinity resins [5, 11–15] and precipitation using magnesium hydroxide [16, 17].

The Institute for Reference Materials and Measurements (IRMM) of the Joint Research Centre (JRC), a Directorate-General of the European Commission, operates the International Measurement Evaluation Program (IMEP). It organises interlaboratory comparisons (ILCs) in support to EU policies. This work presents the outcome of IMEP-40, a PT organised for the determination of 12 trace elements in seawater in support to the Water Framework Directive 2000/60/EC [1]. This PT was carried out under ISO 17043 accreditation [19]. According to this standard, proficiency testing is defined as “the evaluation of participant performance against pre-established criteria by means of interlaboratory comparisons including single occasion exercises – where the proficiency test items are provided on a single occasion”. The IMEP-40 PT belongs to this category of single occasion exercises. The aim of this PT was to assess the performance of laboratories worldwide in the determination and quantification of trace elements in seawater. The study included 12 trace elements (As, Cd, Co, Cr, Cu, Fe, Mn, Mo, Ni, Pb, Se and Zn) present at natural levels in a seawater sample.

## Materials and methods

### Announcement of the study

The PT study was announced on the JRC website and via the European Cooperation for Accreditation (EA), the Asia Pacific Laboratory Accreditation Cooperation (APLAC) and the InterAmerican Accreditation Cooperation (IAAC).

### Preparation and evaluation of the test item

The test material was a candidate Certified Reference Material (CRM) and was produced by IRMM. The raw material for the seawater-based candidate CRM was collected at the Southern Bight off the coast of Belgium (North Sea).

On arrival at IRMM, the three tanks with seawater were placed in a refrigerated container at 4 °C and acidified to pH < 2 with ultrapure hydrochloric acid. The addition of HCl was necessary to ensure stability of the trace element concentrations in the test material over the length of the PT exercise. After acidification, the sample was filtered through a Versaflow 0.8-/0.45-μm filter capsule (PALL, VWR, Belgium). The three vessels with filtered water were left to rest for 4 months in a cooled storage container at 4 °C.

After these 4 months, the seawater was homogenised by recirculation between holding tanks for several working days corresponding to about 40 full mixing cycles in total. Half-way through homogenisation, the seawater-based material was spiked with Cd, Cr, Ni and Zn. Liquid reference standards (1000 mg/L, Merck) were used for this purpose. After spiking, recirculation/homogenisation was carried out for another 20 cycles.

Units of 500 ml seawater were filled in high-density polyethylene (HDPE) bottles with polypropylene (PP) closure. These bottles were acid washed with 2 % nitric acid, rinsed twice with purified water (18.2 MΩ cm^−1^) and dried in a clean cell with high-efficiency particulate arrestance (HEPA) filtered air. The units were labelled according to fill-order. After bottle 0792 was filled, samples for IMEP-40 were filled in every fifth bottle and also labelled according to fill-order.

### Homogeneity and stability

As the test item was a candidate CRM, homogeneity and stability studies were performed in line with the ISO Guide 35 [20]. Short-term stability data were used and expanded to cover the time between dispatch of the samples and reporting of results (8 weeks).

### 2.4 Assigned values and their uncertainties

The assigned values were determined during the certification study of the candidate CRM by a number of expert laboratories (characterisation). Not all expert laboratories reported results for all the analytes. The results of at least three expert laboratories were taken in order to assign the reference values (*X*_ref_) in this PT. For Se, a high variability was observed for both the group of the expert laboratories and the participants in the IMEP-40 study, and therefore, the results for this trace element were not scored. The assigned values, *X*_ref_, for the other trace elements are shown in Table 2.

**Table 2:** Assigned values (*X*_ref_), associated uncertainties (*u*_ref_) and uncertainty contributions (*u*_char_, *u*_bb_, *u*_st,8weeks_). All values are expressed in micrograms per litre. The expanded uncertainty (*U*_ref_) is calculated with a coverage factor *k* = 2 corresponding to a level of confidence of about 95 %

Element	*X*_ref_	*u*_char_	*u*_bb_	*u*_st,8weeks_	*u*_ref_	*U*_ref_
As	1.89	0.051	0.020	0.062	0.083	0.17
Cd	0.096	0.005	0.001	0.004	0.007	0.013
Co	0.075	0.003	0.001	0.005	0.006	0.012
Cr	0.28	0.028	0.003	0.010	0.030	0.06
Cu	0.88	0.034	0.051	0.046	0.076	0.15
Fe	3.5	0.281	0.109	0.134	0.330	0.7
Mn	2.46	0.033	0.020	0.063	0.074	0.15
Mo	12.1	0.342	0.034	0.083	0.354	0.7
Ni	1.06	0.048	0.010	0.030	0.057	0.11
Pb	0.097	0.004	0.003	0.005	0.007	0.014
Zn	4.7	0.121	0.070	0.225	0.265	0.5

The standard uncertainties (*u*_ref_) of the assigned values were calculated combining the uncertainty of the characterisation (*u*_char_) with the contributions for homogeneity (*u*_bb_) and stability (*u*_st_) in compliance with ISO Guide 35 [20] using Eq. 1:

1 $$ {u}_{\mathrm{ref}}=\sqrt{u_{\mathrm{char}}^2+{u}_{\mathrm{bb}}^2+{u}_{\mathrm{st}}^2} $$

The *u*_char_ was calculated according to ISO Guide 35 [20]:

2 $$ {u}_{\mathrm{char}}=\frac{s}{\sqrt{p}} $$

where *s* refers to the standard deviation of the mean values obtained by the expert laboratories and *p* refers to the number of expert laboratories.

Table 2 presents the assigned values (*X*_ref_), the associated uncertainties (*u*_ref_) and uncertainty contributions related to the characterisation, homogeneity and stability (*u*_char_, *u*_bb_, *u*_st,8weeks_) for all elements, except Se, expressed in micrograms per litre. The expanded uncertainty (*U*_ref_) is calculated with a coverage factor *k* = 2 corresponding to a level of confidence of about 95 %.

## Results and discussion

### Scores and their evaluation criteria

Individual laboratory performance was expressed in terms of *z* and *ζ* scores in accordance with ISO 13528 [21]:

3 $$ z=\frac{{\mathrm{x}}_{\mathrm{lab}}-{\mathrm{x}}_{\mathrm{ref}}}{\overset{\frown }{\sigma }} $$

4 $$ \zeta =\frac{{\mathrm{x}}_{\mathrm{lab}}-{\mathrm{x}}_{\mathrm{ref}}}{\sqrt{u_{\mathrm{ref}}^2+{u}_{\mathrm{lab}}^2}} $$

where *X*_lab_ is the measurement result reported by a participant, *u*_lab_ is the standard measurement uncertainty reported by a participant, *X*_ref_ is the assigned value, *u*_ref_ is the standard uncertainty of the assigned value and $\widehat{\upsigma}$ is the standard deviation for proficiency assessment. The measurement results were usually expressed in micrograms per litre. One laboratory reported results in micrograms per kilogram. These results were converted into micrograms per litre using a density of 1.02352 g mL^−1^ which was determined for this candidate CRM. Three laboratories reported “0” values for some elements. These “0” values were not included in the evaluation for *z* and *ζ* scores.

The interpretation of the *z* and *ζ* score was done according to ISO 17043 [19], with |score| ≤2 for a satisfactory performance, 2< |score| <3 for a questionable performance and |score| ≥3 for an unsatisfactory performance.

The *z* score compares the participant’s deviation from the assigned value with the standard deviation for proficiency assessment ($\widehat{\upsigma}$) used as common quality criterion. $\widehat{\upsigma}$ is defined by the PT organiser as the maximum acceptable standard uncertainty. On the basis of previous experience in PTs supporting the EU Water Framework Directive, the standard deviation for the proficiency assessment, $\widehat{\upsigma}$, was set at 25 % of the respective *X*_ref_ for all elements in this IMEP-40 PT study.

The *ζ* score is useful to check if the result of a participant is close to the assigned value within its claimed measurement uncertainty. An advantage of the *ζ* score is that the complete result of the participant, including its measurement uncertainty is assessed against the assigned value, its uncertainty and the unit of the result. An unsatisfactory performance expressed as *ζ* score may therefore indicate a large difference between *X*_lab_ and *X*_ref_, an underestimation of the measurement uncertainty by the participant or a combination of both.

The expanded uncertainty reported by the participant was divided by the reported coverage factor, *k*, to calculate the standard uncertainty of the participant (*u*_lab_). In case participants did not report their measurement uncertainty, it was put at zero (*u*_lab_ = 0). When participants did not specify *k*, the reported expanded uncertainty was considered as the half-width of a rectangular distribution and *u*_lab_ was then calculated by dividing this half-width by √3, following the recommendation of Eurachem and CITAC [22–24].

### Laboratory results and scoring

Forty-six laboratories from 26 different countries (Argentina, Bosnia-Herzegovina, Brazil, Bulgaria, Canada, Columbia, Costa Rica, Denmark, Ecuador, El Salvador, Finland, France, F.Y.R. of Macedonia, Germany, Ireland, Italy, Latvia, The Netherlands, Philippines, Poland, Qatar, Spain, Sweden, Switzerland, UK, USA) reported results in this exercise. Of these 46 laboratories, 34 were commercial laboratories, 11 governmental laboratories and 1 was a university laboratory. The laboratories received a list of the elements present in the sample, but not all laboratories reported results for all elements. Table 3 shows that the number of results reported for the different elements ranged from 36 (Mo) to 44 (Cu, Ni, Pb), including the “less than X” values.

**Table 3:** Total number of reported results, number of reported values, number of reported “less than X” values and number of correct (*X* ≥ *X*_ref_ − U_ref_) and incorrect (*X* < *X*_ref_ − *U*_ref_) “less than X” values for each element

Analyte	Number of reported results	Number of reported values	Number of “less than X” values	Correct “less than X” values	Incorrect “less than X” values
As	43	36	7	4	3
Cd	43	25	18	16	2
Co	40	24	16	16	0
Cr	41	23	18	18	0
Cu	44	31	13	13	0
Fe	43	27	16	16	0
Mn	43	37	6	4	2
Mo	36	29	7	3	4
Ni	44	33	11	11	0
Pb	44	21	23	23	0
Se	37	20	17	–	–
Zn	43	33	10	10	0

Many of the elements were present at low concentrations equal to natural levels. This resulted in a range of 14.0 % (Mn) to 52.3 % (Pb) of laboratories reporting “less than X” values for the different elements. The limit values “X” reported by the laboratories usually correspond to the limits of quantification (LOQ) or limits of detection (LOD) of the applied methods. Those reporting “less than X” values were not included in the evaluation but the reported “less than X” values were compared with the corresponding *X*_ref_ − *U*_ref_. If the reported limit value “X” is lower than the corresponding *X*_ref_ − *U*_ref_, the statement “less than X” is considered incorrect, since the laboratory should have been able to quantify or detect the respective element. The number of correct and incorrect “less than X” values is summarised in Table 3. It can be observed that for 7 out of the 11 scored trace elements, all laboratories made a correct statement, meaning that the amount of the element present in the seawater was actually below the LOQ or LOD of their method.

**Fig. 1:**
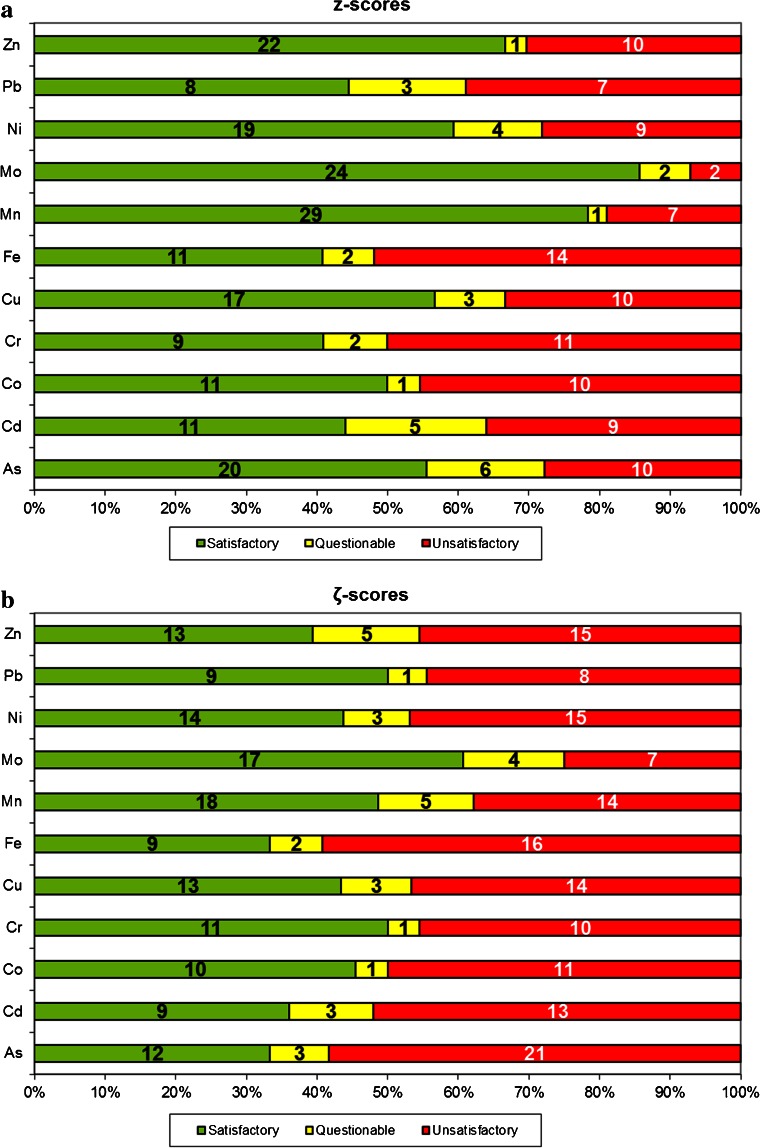
Number of evaluated laboratories with satisfactory, questionable and unsatisfactory *z* scores (**a**) and *ζ* scores (**b**). (The *numbers* on the *bars* correspond to the exact number of laboratories in a certain scoring category)

An overview of the obtained *z* scores and *ζ* scores is presented in Fig. 1: the percentages of satisfactory performances expressed as *z* score (|*z*| ≤ 2) for the 11 evaluated elements ranged from 41 % (Cr, Fe) to 86 % (Mo), while the satisfactory performances expressed as *ζ* scores (|*ζ*| ≤ 2) ranged from 33 % (As, Fe) to 61 % (Mo) of the participants. The percentages of unsatisfactory performances (|*z*| ≥ 3) ranged from 7 % (Mo) to 52 % (Fe). When looking in detail to the reported results (PT report to participants [25]), it was observed that 87.9 % of these unsatisfactory performances (|*z*| ≥ 3) was caused by an overestimation of the amounts of trace elements. This may be due to polyatomic interferences as presented in Table 1. However, in some cases, the overestimation reached a factor of 100 and more. For some elements, such as As and Fe, this can be caused by polyatomic interferences. The result for As may be high because of ArCl^+^ interference, while for Fe, it may be high because laboratories did not resolve the ArO^+^ interference. Considering the low levels of trace elements in this test material, contamination was also a risk for elements more commonly found in the environment such as Fe or Zn.

**Fig. 2:**
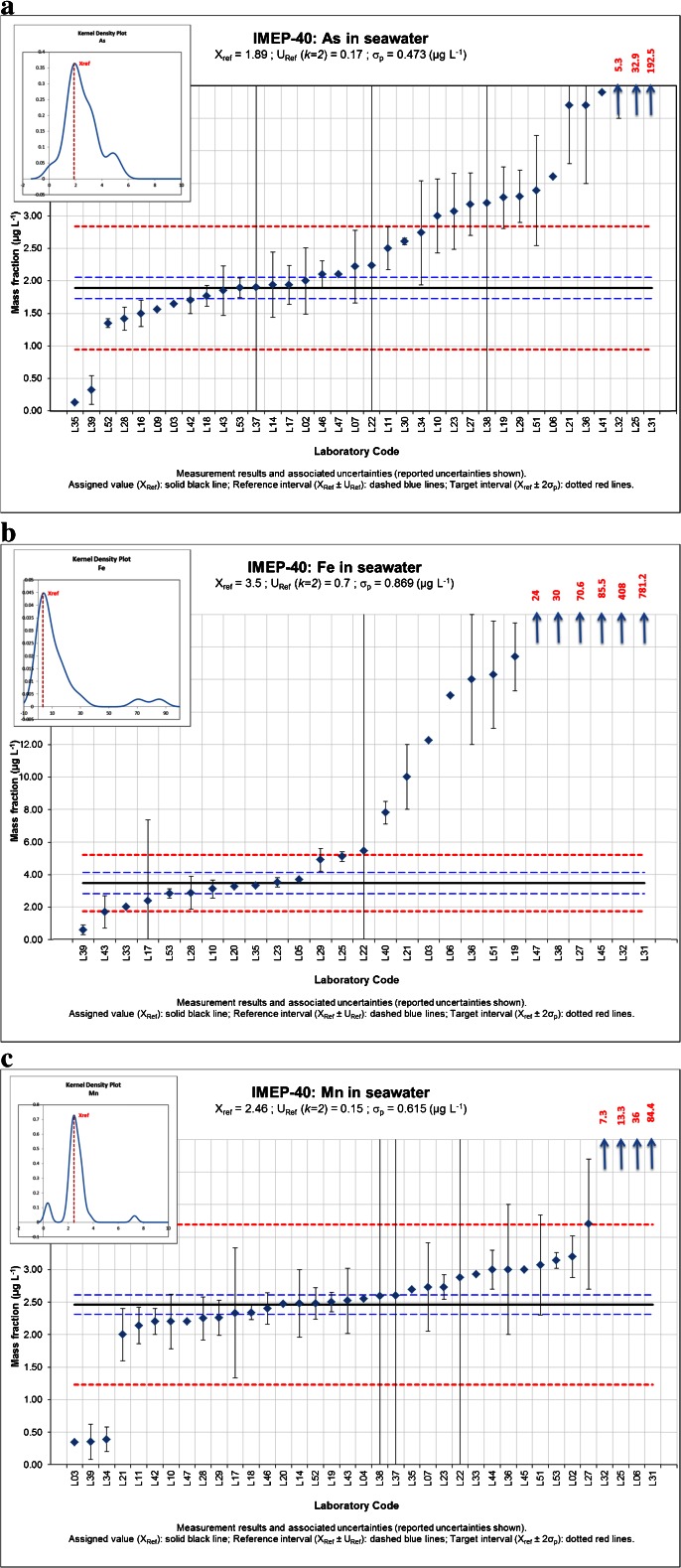
Participant results for As (**a**), Fe (**b**) and Mn (**c**)

In order to illustrate some data more in detail, results for three representative trace elements (As, Fe, Mn) are shown in Fig. 2. The graph displays the measurement results and associated expanded uncertainties of the participants and the assigned value *X*_ref_ with a reference interval (*X*_ref_ ± *U*_ref_) and a target interval (*X*_ref_ ± 2$\widehat{\upsigma}$). Taking into account Eq. 3, this target interval includes all satisfactory performances (|*z*| ≤ 2). In the graph, *σ*_p_ stands for $\widehat{\upsigma}$. Furthermore, it includes a Kernel density plot which gives the probability density function of all reported measurement results together with the assigned value *X*_ref_. The Kernel density plot is used to check if there is a distribution different from normal of the measurement results (>1 major peak). In this exercise, a bimodal or even a multimodal distribution was found for As (Fig. 2) and for some of the other elements.

**Fig. 3:**
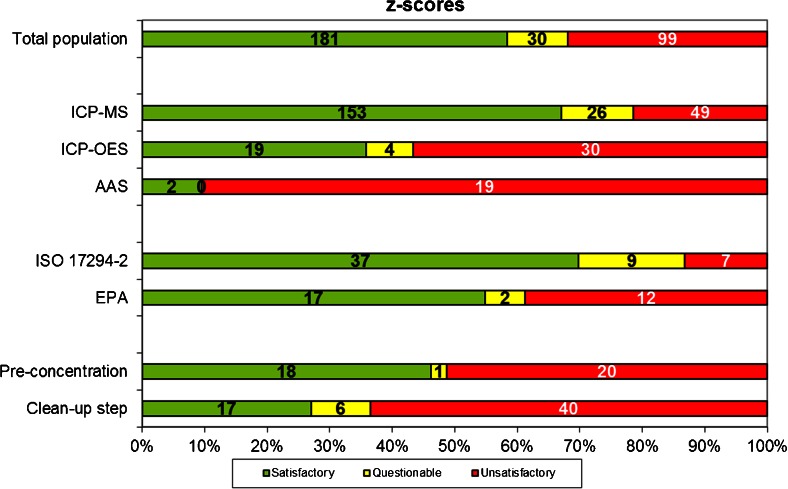
Total number satisfactory, questionable and unsatisfactory *z* scores obtained with different methodologies and detection techniques. (The *numbers* on the *bars* correspond to the total number of *z* scores for all elements in a certain scoring category)

The techniques used for the measurement of the different elements are summarised in Table 4. ICP-MS was the most common technique, followed by ICP-OES. AAS, comprising flame AAS, ET-AAS and the single-element technique hydride generating atomic absorption spectroscopy (HG-AAS), was used to a lesser extent. Some techniques were used in only a few measurements: total reflection X-ray fluorescence (TXRF) and the single-element techniques atomic fluorescence spectroscopy (AFS), UV-VIS and colorimetry. Table 4 also summarises the number of “less than X” values reported per technique. It can be observed that for the three most widely applied techniques (ICP-MS, ICP-OES, AAS), ICP-OES gives the highest percentage of “less than X” values (50.0 %), followed by AAS (36.4 %) and ICP-MS (25.6 %). This is a consequence of the fact that without sample pre-concentration, the LODs for ICP-OES-based techniques are likely to be higher than those of the other techniques. Even though AAS seems to perform better than ICP-OES in this respect, AAS led to a high percentage of unsatisfactory performances (|*z*| ≥ 3), as can be observed in Fig. 3. Only 2 out of the 21 reported results with AAS showed a satisfactory performance (|*z*| ≤ 2). Moreover, these two results were both obtained for As using HG-AAS. Therefore, it can be concluded that AAS without hydride generation is less suitable for the analysis of low level trace elements in seawater. With ICP-OES, 36.5 % of satisfactory performances (|*z*| ≤ 2) were obtained. This observation together with the high number of “less than X” values seems to indicate that also this technique is not the most appropriate for the analysis of low level trace elements in seawater. Best results were obtained with ICP-MS leading to 67.1 % of satisfactory performances (|*z*| ≤ 2) and 21.5 % of unsatisfactory performances (|*z*| ≥ 3).

**Table 4:** Techniques used expressed as total number of measurements (% are given for three most used techniques and are relative to total number of measurements with all techniques in column 1 and relative to total number of measurements with corresponding technique in column 2)

	Number of measurements	Number of “less than X” values
ICP-MS	305 (67.2 %)	78 (25.6 %)
ICP-OES	106 (23.2 %)	53 (50.0 %)
AAS	33 (7.3 %)	12 (36.4 %)
TXRF	6	0
AFS	2	1
UV-VIS	1	1
Colorimetry	1	0

**Fig. 4:**
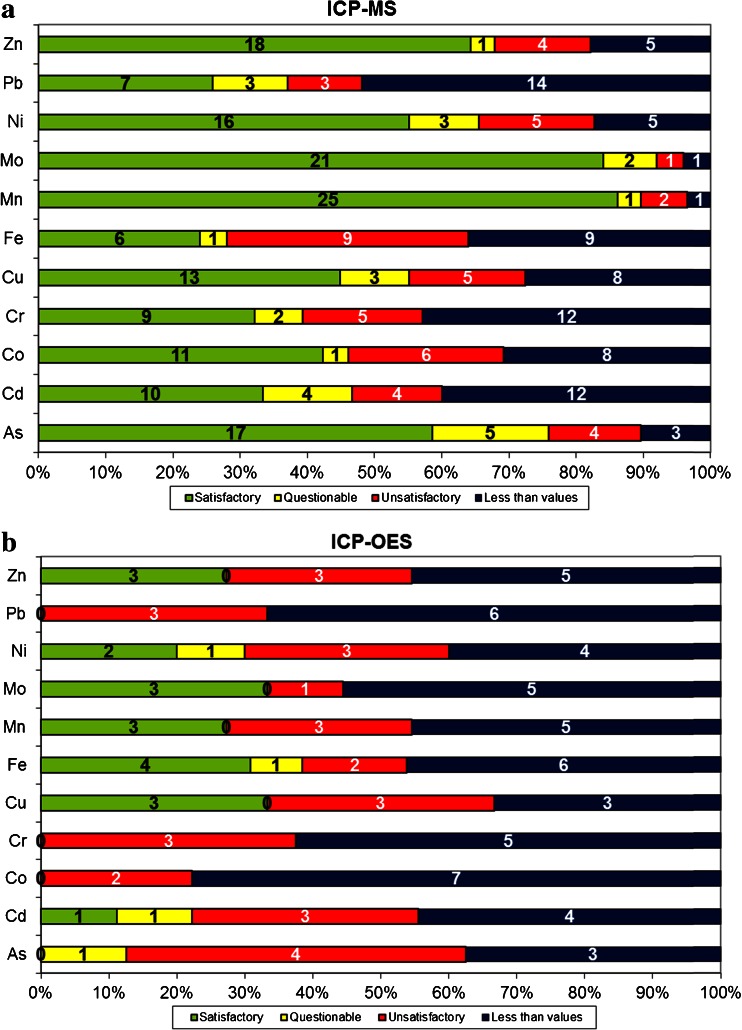
Number of laboratories with satisfactory, questionable and unsatisfactory *z* scores and number of laboratories with “less than X” values per element and using ICP-MS (**a**) or ICP-OES (**b**). (The *numbers* on the *bars* correspond to the exact number of laboratories in a certain scoring category)

As the LODs and interferences vary between the elements depending on the technique used, the performances obtained with ICP-MS and ICP-OES were split up per element in Fig. 4 in order to distinguish element-dependent performances for both techniques. For ICP-MS, the best performances were obtained for Mo and Mn with high rates of satisfactory performances (|*z*| ≤ 2) and only one reported “less than X” value for each element. For Pb, the low concentration level in the seawater sample (0.097 μg L^−1^) leads to a high number of “lower than LOD/LOQ” values. Notwithstanding, seven of the eight satisfactory results were generated by ICP-MS, indicating its suitability for low level measurement. On the other hand, ICP-MS seemed less suitable for Fe analysis. Fe showed an equally high number of unsatisfactory performances (|*z*| ≥ 3) as “less than X” values. Moreover, when looking at the results obtained for Fe with ICP-OES in Fig. 4b, it can be observed that ICP-OES performed better than ICP-MS for this element. Nevertheless, Fe seemed to be the exception in this respect, which is likely due to the strong isobaric interference of ArO^+^ ions on Fe measurement by ICP-MS. In contrast, ICP-MS showed better performance for As in spite of the potential for ArCl^+^ interference on seawater analysis. While none of the few ICP-OES measurements returned a satisfactory result for As, 58.6 % of ICP-MS results met this target. No satisfactory performances (|*z*| ≤ 2) were obtained when ICP-OES was used for the analysis of As, Co, Cr and Pb and for all other elements, except Fe, the rates of satisfactory performances were lower for ICP-OES than for ICP-MS.

Single-element techniques were used the most for the analysis of As: besides the two laboratories using HG-AAS, two laboratories mentioned the use of AFS and one laboratory the use of colorimetry. UV-VIS was used by one laboratory for the analysis of Fe but the LOD of this method was too high.

The low concentration levels of the trace elements in a difficult matrix (high saline content) need to be taken into consideration to understand the relatively low rate of satisfactory performances in this PT exercise. Laboratories showing a systematic positive bias were advised to evaluate their methods in order to exclude any kind of interferences or contamination.

### Questionnaire results

Participants were asked to fill in a questionnaire with the aim of gathering information about the laboratories and the analytical methods used. Thirty-eight laboratories filled in the associated questionnaire. According to those responses, 19 participants used a standardised method while 19 did not. The standard method which was used the most (by six labs) was the ISO 17294-2 “Water quality—Application of inductively coupled plasma mass spectrometry (ICP-MS)—Part 2: Determination of 62 elements”. A number of laboratories used one of the methods of the US Environmental Protection Agency: three laboratories applied the EPA 6020A method (ICP-MS, water and solid waste), one the EPA 6010C method (ICP-AES) and two the EPA 200.8 method (ICP-MS, water and wastewater). Other methods used were the Standard Methods for the Examination of Water and Wastewater (SMEWW) part 3120 B (two labs), the ISO 11885:2009 “Water quality. Determination of selected elements by inductively coupled plasma optical emission spectrometry (ICP-OES)” (one lab), APHA 3125 “Metals by Inductively Coupled Plasma/Mass Spectrometry” (one lab) and APHA 3111C “Metals by Flame Atomic Absorption Spectrometry” (one lab). Two labs mentioned the use of an official method without further specifications. Figure 3 shows the overall performance when applying the ISO 17294-2 and the EPA methods. The best overall performance was obtained with the ISO 17294-2 method, leading to 69.8 % of satisfactory performances (|*z*| ≤ 2). This can be linked to the performance obtained with ICP-MS (67.1 % of satisfactory performances). However, the percentage of unsatisfactory performances (|*z*| ≥ 3) with the ISO 17294-2 method further decreased to 13.2 % (compared to 21.5 % with ICP-MS) and the number of “less than X” values decreased to 19.7 % (compared to 25.6 % with ICP-MS). The performance with the EPA methods was in line with the performance seen in the total population (Fig. 3).

Surprisingly, only a minority of the laboratories used a clean-up step (eight laboratories) or a pre-concentration technique (six laboratories). Figure 3 shows that none of these two steps seemed to contribute to a better performance: laboratories using pre-concentration only obtained 46.2 % of satisfactory performances (|*z*| ≤ 2) while laboratories using a clean-up step only obtained 27.0 % satisfactory performances (|*z*| ≤ 2). It has to be remarked that these low ranges of satisfactory performances may not be caused by these sample preparation techniques directly but by the instrumental techniques coupled to them: in many cases, not ICP-MS but ICP-OES and AAS were combined with them. One laboratory using pre-concentration combined with TXRF obtained a satisfactory performance (|*z*| ≤ 2) for the six elements it analysed.

Only one laboratory managed to analyse all 11 scored elements satisfactorily. According to the questionnaire, this laboratory used ICP-MS, without clean-up step or pre-concentration technique and without using an official method. It used CRMs (NRCSLRS-5, NWTM27.3, NIST-1640a) in order to validate its method and correct the results for recovery. Only 31.6 % of the laboratories corrected their results for recovery. However, in general, no correlation was found between the correction for recovery and the performance in the PT.

To the question whether the participants usually provide an uncertainty estimate to their customers, half of the laboratories (19 out of 38) replied they do. In this PT, most participants provided an uncertainty estimation of their results. The following different approaches were used: uncertainty budget (ISO-GUM), uncertainty of the method (in-house validation), measurement of replicates (precision), estimation based on judgement, the use of inter-comparison data and the use of the ISO 11352 standard (Water quality. Estimation of measurement uncertainty based on validation and quality control data). In many cases, laboratories combined two or more approaches to make an uncertainty statement. The most frequent used approaches were the uncertainty estimation based on results obtained during the in-house validation (23 laboratories) or based on the measurement of replicates (20 laboratories). The latter approach may result in an underestimation of the measurement uncertainty and explain why for most of the elements the number of satisfactory performances expressed as |*ζ*| ≤ 2 is lower than the number of satisfactory performances expressed as |*z*| ≤ 2 (Fig. 1). Indeed, according to Eq. 4, an underestimated *u*_lab_ will result in an increased |*ζ* score|. A second cause of increased |*ζ* scores| may be the fact that a number of laboratories did not report an uncertainty estimate in which case it was set to zero (*u*_lab_ = 0). The underestimation of the measurement uncertainty by some laboratories can also be observed in Fig. 2. In the three graphs, results can be found for which the associated uncertainty interval does not include the assigned value. When these results lay within the target interval, they have a |*z*| ≤ 2, but due to the underestimated measurement uncertainty, typically a |*ζ*| > 2. Some laboratories also overestimated their measurement uncertainty, although in some cases, this was caused by the use of a wrong unit (e.g. %).

## Conclusions

The analysis of natural levels of trace elements in seawater was challenging for the laboratories participating in IMEP-40. The low concentration levels of the trace elements combined with the high saline content of the seawater resulted in a high number of laboratories unable to detect or quantify the elements. When reporting, a relatively low number of laboratories showed a satisfactory performance mostly due to overestimation of the amounts of elements in the seawater. The PT study showed that the use of proper standard methods, like ISO 17294-2, and sensitive techniques, like inductively coupled plasma mass spectrometry (ICP-MS), contributed to achieve a good performance.
